# Diffuse Large B-Cell Lymphoma With Tracheoesophageal Fistula Healed Using Chemotherapy: A Case Report and Review of the Literature

**DOI:** 10.7759/cureus.78959

**Published:** 2025-02-13

**Authors:** Naoko Yagi, Takuro Yoshimura, Minako Tsutsumi, Takafumi Nakao

**Affiliations:** 1 Hematology, Osaka City General Hospital, Osaka, JPN; 2 Hematology, Osaka Metropolitan University Graduate School of Medicine, Osaka, JPN

**Keywords:** aerodigestive fistula, bronchoesophageal fistula, diffuse large b-cell lymphoma, esophagorespiratory fistula, lymphoma, tracheoesophageal fistula

## Abstract

Malignant lymphomas are rarely associated with tracheoesophageal or bronchoesophageal fistulas (TEFs/BEFs). Lymphomas accompanied by TEFs/BEFs have a more favorable prognosis than solid tumors. Here, we present a unique case of a 74-year-old patient with diffuse large B-cell lymphoma (DLBCL). Contrast-enhanced computed tomography (CT) revealed TEF, aspiration pneumonia, and multiple lymphadenopathy. Histopathology helped confirm DLBCL, leading to a Lugano IIE staging. Complete DLBCL remission could be achieved by adjusting chemotherapy dosages for complications, which also resulted in spontaneous TEF resolution without requiring interventions such as stent insertion or surgical correction. No instances of lymphoma recurrence or tracheoesophageal fistula were observed 46 months post-chemotherapy. Among 33 documented cases, this is the first instance of DLBCL-associated TEF resolution through chemotherapy. Notably, in patients initially presenting with a fistula prior to treatment initiation, a favorable response to chemotherapy and/or radiotherapy coupled with controlled aspiration could potentially lead to the resolution of the fistula. Collectively, our case highlights the potential for conservative fistula management and the possibility of spontaneous resolution with chemotherapy. Our report provides valuable insights into lymphoma-associated fistulas and their management.

## Introduction

Diffuse large B-cell lymphoma (DLBCL) is an aggressive and the most common subtype of non-Hodgkin lymphoma (NHL), accounting for 30-40% of cases. Tracheoesophageal or bronchoesophageal fistulas (TEFs/BEFs) are pathological connections between the tracheobronchial tree and the esophagus. These fistulas typically present with symptoms such as dysphagia, fever, weight loss, and the characteristic sign of coughing during meals [1]. While malignant esophagorespiratory fistulas are frequently associated with esophageal and lung cancers, with squamous cell carcinoma (SCC) accounting for 85.5% to 87.9% of cases, their occurrence in lymphoma is rare, constituting merely 0.4% to 1.4% of all cases [2,3]. Solid tumor-associated TEFs/BEFs have a poor prognosis, characterized by a median survival duration of 35 days [3,4]. Conversely, lymphoma-related TEF/BEF exhibits a relatively favorable prognosis relative to solid tumors [5-8]. Limited reports, particularly those involving Hodgkin's lymphoma and low-grade NHL, have documented the healing of fistulas subsequent to chemo/radiotherapy without the need for surgical or stenting interventions. However, this phenomenon has not been reported in patients diagnosed with diffuse large B-cell lymphoma (DLBCL). We present the first case of DLBCL, an aggressive NHL, with TEF resolution following chemotherapy alone.

## Case presentation

**Figure 1:**
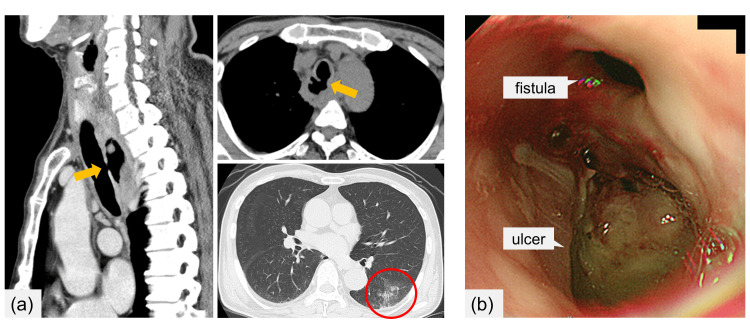
Computed tomography (CT) image and upper gastrointestinal endoscopy findings upon admission (a) CT image depicted a tracheoesophageal fistula measuring 5 mm in diameter and 1 cm in length, alongside aspiration pneumonia. (b) Upper gastrointestinal endoscopy findings revealed an ulcerous lesion and a fistulous connection to the trachea within the cervical esophagus.

**Figure 2:**
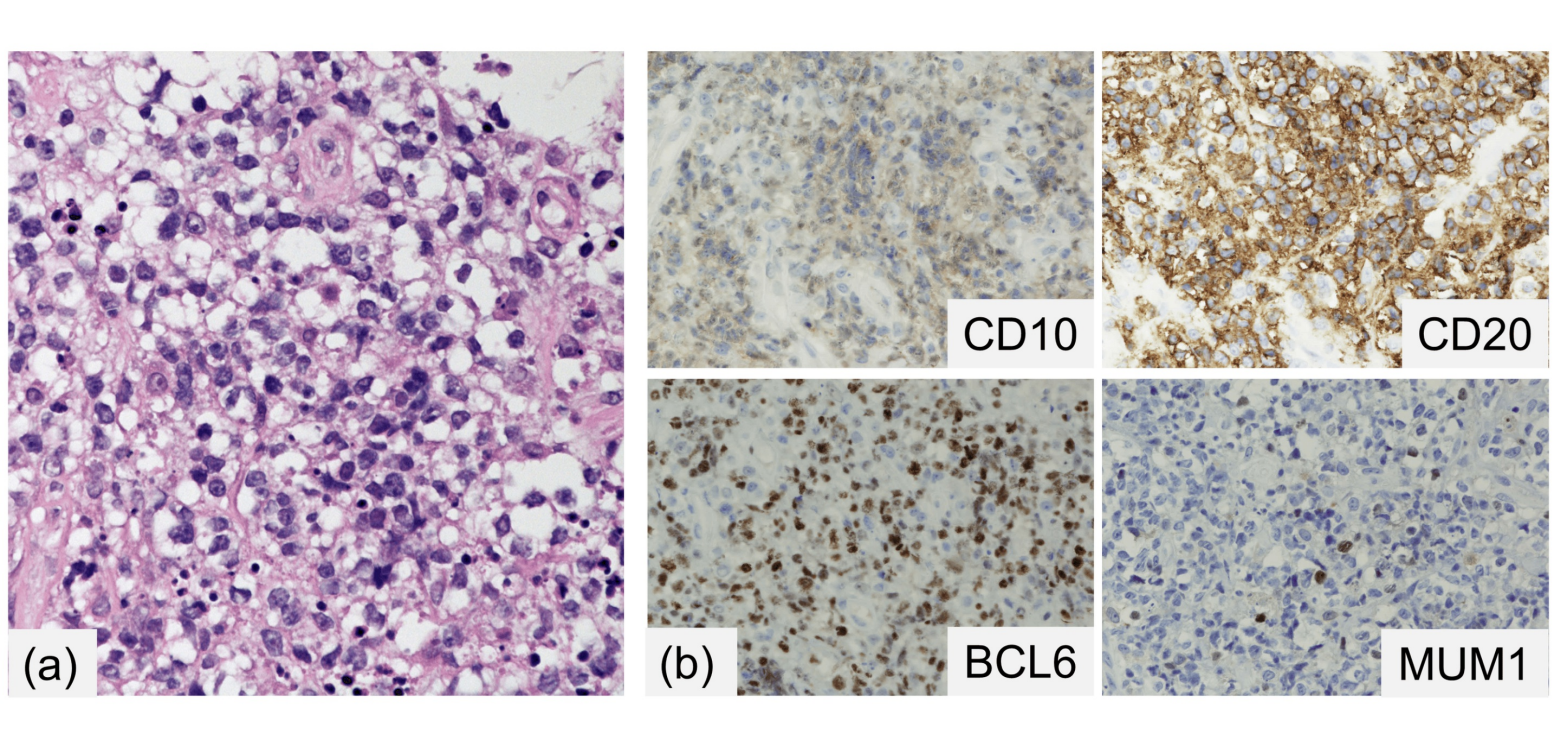
Histopathological findings of the right supraclavicular lymph node (a) Hematoxylin-eosin staining showed the diffuse infiltration of abnormal large lymphoid cells (at 400× magnification). (b) Immunostaining indicated the positive staining of abnormal lymphoid cells for CD20 and BCL6 while testing negative for CD10 and MUM1.

A 74-year-old man with a history of hypertension and prostate cancer was referred to our hospital with a 2-week history of worsening productive cough and fever. Contrast-enhanced computed tomography (CT) revealed indications of right supraclavicular and mediastinal lymphadenopathy alongside a cervical-to-thoracic esophageal mass. Additionally, CT indicated a TEF (measuring 5 mm in diameter and 1 cm in length) and signs of aspiration pneumonia (Figure 1a). Upper gastrointestinal endoscopy revealed an ulcer and a tracheal fistulous connection at the site of cervical esophageal stenosis (Figure 1b). Histopathological analysis demonstrated primarily necrotic esophageal ulcers. Furthermore, a right supraclavicular lymph node biopsy revealed diffuse infiltration of abnormally large lymphoid cells, demonstrating positive CD20 and BCL6 expression and negative CD10 and MUM1 expression (Figure 2). The patient was diagnosed with DLBCL (germinal center B-cell type), with the bone marrow remaining unaffected, resulting in classification under the Lugano IIE clinical stage.

**Figure 3:**
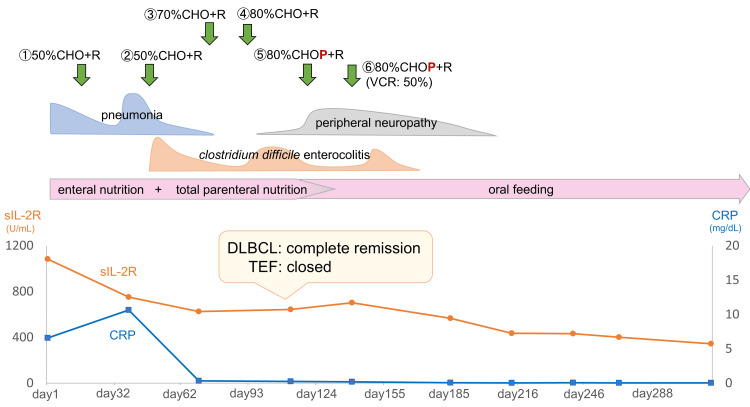
Clinical course C: cyclophosphamide; H: daunorubicin; O, VCR: vincristine; P: prednisolone; R: rituximab; sIL-2R: soluble interleukin-2 receptor; CRP: C-reactive protein

**Figure 4:**
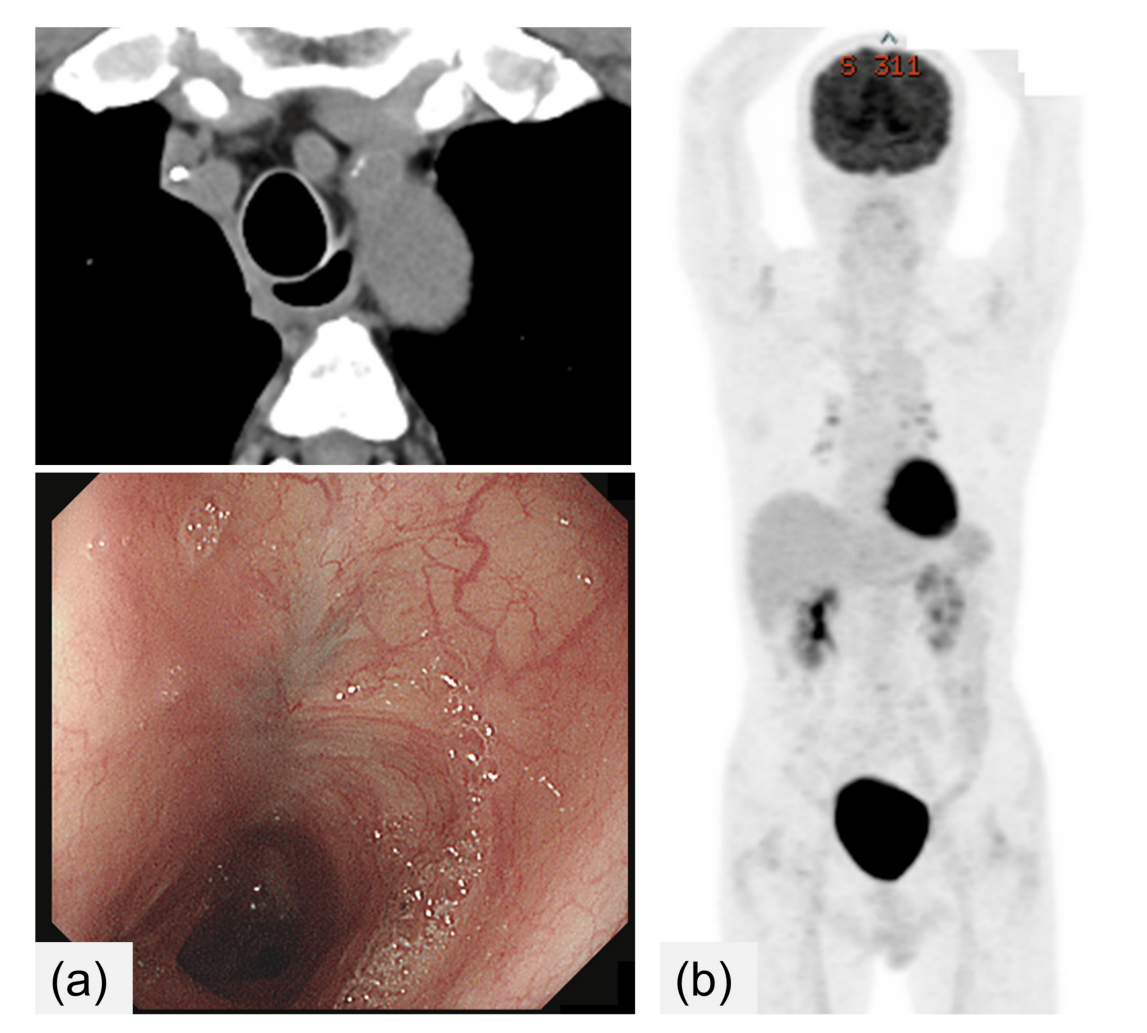
Images after chemotherapy (a) CT and endoscopy findings after four chemotherapy cycles showed fistula closure and complete remission of DLBCL. (b) Positron emission tomography (PET) image after six chemotherapy cycles indicated complete metabolic remission.

The aspiration pneumonia was managed with antibiotics, oral intake cessation, and initiation of enteral nutrition via a gastric tube. After successful pneumonia control, a regimen of reduced-dose rituximab-combined chemotherapy was initiated (cyclophosphamide 375 mg/m^2^, doxorubicin 25 mg/m^2^, and vincristine 0.7 mg/m^2^ administered intravenously on day 1, and rituximab 375 mg/m^2^ administered intravenously on day 7). Owing to concerns about exacerbating pneumonia, steroids were excluded from this regimen. Notably, pneumonia recurred post-initial chemotherapy owing to vomitus aspiration. Subsequent to the resolution of pneumonia with antibiotics, the second chemotherapy cycle was initiated using the same regimen alongside total parenteral nutrition. From the third cycle onward, the chemotherapeutic dosage was progressively increased, with a total of six cycles administered (Figure 3). The patient achieved complete remission of DLBCL after four cycles, along with fistula closure (Figure 4). No esophageal leaks were observed during esophageal angiography. As the risk of aspiration diminished, steroids (prednisolone 100 mg/body, orally administered on days 1-5) were introduced during the fifth and sixth chemotherapy cycles. Following the fourth cycle, a liquid diet was reintroduced, eventually progressing to a regular diet 6 months post-chemotherapy completion. The patient presently maintains a well-nourished status through oral feeding. As of 46 months post-chemotherapy completion, there have been no observed instances of lymphoma recurrence or TEF.

## Discussion

To the best of our knowledge, this is the first reported case of DLBCL-associated TEF that healed following chemotherapy. Our study presents two critical insights. First, in lymphoma-related TEF/BEF, minimizing the risks of aspiration and infection is paramount, requiring a preliminary conservative approach for fistula management. Second, in cases that initially present with a fistula followed by a positive response to chemotherapy and/or radiotherapy, there is a possibility of spontaneous fistula resolution without the requirement for interventions, such as stenting and surgical correction.

Between 1990 and 2023, we identified 33 documented instances of lymphoma complicated by TEF/BEF within the English literature (Table 1) [6-39]. The median age in these cases was 53 years (range: 10-79 years), with 18 men and 15 women. Notably, Hodgkin's lymphoma emerged as the most prevalent subtype (15/33 cases, 45%). Within non-Hodgkin's lymphomas, DLBCL constituted the predominant type (7/33 cases, 21%).

**Table 1:** Thirty-three cases of malignant lymphoma with tracheo/bronchoesophageal fistula reported in the English literature Thirty-three cases of malignant lymphoma with tracheo/bronchoesophageal fistula were reported in English language publications between 1990 and 2023, as identified by searching the keywords “tracheoesophageal fistula” OR “bronchoesophageal fistula” OR “aerodigestive fistula” OR “esophagorespiratory fistula” AND “lymphoma” on PubMed and Google Scholar. M: male; F: female; most: months; yrs: years; OTSC: over-the-scope clip; NHL: non-Hodgkin's lymphoma; HL: Hodgkin's lymphoma; ALCL: anaplastic large cell lymphoma; PTCL-NOS: peripheral T-cell lymphoma, not otherwise specified; PMBL: primary mediastinal B-cell lymphoma; MALT: mucosa associated lymphoid tissue; N/A: not available; chemo: chemotherapy; RT: radiotherapy; CR: complete response; PR: partial response; ChlVPP: chlorambucil-vinblastine-prednisolone-procarbazine; CMOP: cyclophosphamide-mitoxantrone-vincristine-prednisone; CY: cyclophosphamide; PSL: prednisolone; ASCT: autologous stem cell transplantation; COP: cyclophosphamide-vincristine-prednisone; AVD: doxorubicin-vinblastine-dacarbazine; BEACOPP: bleomycin-etoposide-doxorubicin-cyclophosphamide-vincristine-procarbazine-prednisone; DXR: doxorubicin; VCR: vincristine; ETP: etoposide; DTIC: dacarbazine; GVD: gemcitabine-vinorelbine-doxorubicin; CVP: cyclophosphamide-vincristine-prednisone; BV: brentuximab vedotin; GDP: gemcitabine-cisplatin-dexamethasone

No.	First Author, Year	Age, Gender	Disease Type	Stage	Fistula Locations	Fistula Size (mm)	Time of Fistula Discovery	Treatment for Lymphoma	Management for Fistula				Outcome	Fistula Closure Status	Follow-up Period
									Surgery	Stent Placement	OTSC	Enteral Nutrition			
1	Sharpe et al., 1992 [17]	55, M	HL	ⅣB	left main bronchus	4	after 4th chemo cycle	chemo	yes	(-)	(-)	(-)	remission	artificial closure	20 mos after initial diagnosis
2	Greven and Evans., 1992 [14]	25, F	HL	ⅡB	trachea	N/A	at relapse	RT	(-)	(-)	(-)	yes	remission	spontaneous closure	7 mos post-RT
3	Alba et al., 1994 [18]	60, F	HL	N/A	right main bronchus	N/A	prior to lymphoma diagnosis	none	(-)	(-)	(-)	(-)	♰ death (respiratory failure)	persistence	7 days post-admission
4	Pac-Ferrer et al., 1994 [19]	15, M	HL	N/A	mid trachea	15	during treatment	chemo, RT	(-)	yes	(-)	(-)	progression	artificial closure	N/A
5	Small et al., 1995 [20]	54, M	HL	IV	left main bronchus	7	at diagnosis	chemo (ChlVPP)	yes	(-)	(-)	(-)	relapse	artificial closure	6 mos post-chemo
6	Lingand Bushunow., 1996 [7]	47, F	HL	ⅡB	mid trachea	N/A	at relapse	chemo, RT	(-)	(-)	(-)	yes	remission	spontaneous closure	1 yr post-chemo
7	Lackner et al., 1996 [8]	32, M	DLBCL	ⅡA	trachea	8 x 6	during chemo	chemo (CMOP)	yes	(-)	(-)	(-)	remission	artificial closure	18 mos post-surgery
8	Zueger et al., 1997 [9]	56, M	low-grade NHL	ⅠB	left main bronchus	6	at diagnosis	chemo (CY/PSL)	(-)	(-)	(-)	yes	remission	spontaneous closure	2 yrs post-chemo
9	Jougon and Couraud., 1998 [21]	19, F	NHL	N/A	trachea	60	2 yrs post-chemo	chemo (CHOP)	yes	(-)	(-)	yes	remission	artificial closure	4 yrs post-surgery
10	Kassis et al., 1998 [22]	70, M	HL	ⅢB	bronchus intermedius	N/A	at diagnosis	chemo (ABVD)	yes	(-)	(-)	(-)	♰ death (lymphoma progression)	artificial closure	8 mos after initial presentation
11	Fan et al., 2002 [23]	58, M	ALCL	N/A	mid trachea	N/A	post-chemo	chemo, RT	(-)	yes (esophagus, trachea)	(-)	(-)	progression	artificial closure	N/A
12	Hosoya et al., 2004 [24]	65, M	DLBCL	Ⅳ	trachea	10	during first CHOP cycle	chemo	yes	(-)	(-)	yes	remission	artificial closure	2 yrs post-surgery
13	Bernal et al., 2005 [25]	33, F	HL	N/A	right main bronchus	25	at diagnosis	chemo	(-)	yes (esophagus)	(-)	yes	CR	artificial closure	N/A
14	Munshi et al., 2006 [12]	27, M	HL	ⅡB	trachea	N/A	at diagnosis	chemo (ABVD), RT	N/A	N/A	N/A	N/A	CR	spontaneous closure	1 yr post-treatment completion
15	Moree et al., 2006 [26]	10, M	T-cell lymphoblastic lymphoma	N/A	trachea	50	during chemo	chemo	(-)	(-)	(-)	yes	♰ death (bleeding)	persistence	66 days post-admission
16	Valenti et al., 2008 [10]	68, M	DLBCL	N/A	trachea	N/A	post-chemo	chemo (R-CHOP)	yes	yes	(-)	yes	remission	artificial closure	N/A
17	Joshi et al., 2008 [15]	56, M	ALK-1 positive ALCL	ⅢB	trachea	N/A	at diagnosis	chemo (CHOP, DHAP), ASCT	(-)	(-)	(-)	yes	good response	spontaneous closure	N/A
18	Kutchuk et al., 2009 [27]	36, F	relapsed HL	Ⅳ	trachea (connected to esophagus and left common carotid artery)	13	post-mortem (autopsy)	none	(-)	(-)	(-)	(-)	♰ death (hemorrhagic shock)	persistence	12 hours post-admission
19	Sharma et al., 2011 [28]	63, M	PTCL-NOS	advanced stage	right intermediate bronchus	N/A	at diagnosis	chemo (COP)	(-)	(-)	(-)	yes	good response	N/A	N/A
20	Westin et al., 2012 [16]	53, F	HL	advanced stage	trachea	N/A	6 yrs post-diagnosis (untreated)	chemo (AVD)	(-)	yes (trachea)	(-)	yes	CR	spontaneous closure (stent later removed)	6 yrs post-initial treatment
21	Rigney et al., 2013 [11]	29, M	HL	ⅢB	right upper lobe bronchus	30	at diagnosis	chemo (BEACOPP)	(-)	yes (esophagus)	(-)	yes	good response	spontaneous closure (stent later removed)	3 mos post-chemo completion
22	So and Adler, 2014 [29]	76, M	NHL	N/A	trachea	10	post-chemo	chemo	(-)	yes (esophagus)	yes	yes	N/A	artificial closure	N/A
23	Munikoty et al., 2017 [13], Sen et al., 2022 [30]	10, F	HL	ⅢBE	mid trachea (two locations)	22 x 25 x 24	at diagnosis	chemo (DXR/VCR/ETP/PSL/CY/DTIC)	yes	(-)	(-)	yes	good response	artificial closure	more than 1 y post-surgery
24	Teerakanoket al., 2017 [6]	60, M	DLBCL	ⅣB	trachea, left main bronchus	30 and 15, in double location	at diagnosis	chemo (R-CHOP)	(-)	yes (esophagus, tracheobronchial tree)	(-)	yes	good response	persistence	until completion of chemo
25	Dai et al., 2017 [31]	52, F	MALT lymphoma	N/A	trachea	6 x 7	6 mos post-tracheal resection (TEF caused by prolonged tracheal stent placement and RT for post-resection stenosis)	surgery (tracheal resection and carina reconstruction)	yes (reoperation)	yes (trachea)	(-)	yes	remission	artificial closure	N/A
26	Kashyap et al., 2019 [32]	21, F	PMBL	N/A	trachea	N/A	post-chemo	RT, chemo (Rituximab, CY/DXR/VCR)	yes	yes (esophagus) → removed	(-)	yes	palliative care	N/A	N/A
27	Smiti et al., 2019 [33]	27, M	HL	IV	right bronchus	N/A	1 m after 2nd chemo cycle	RT, chemo	(-)	(-)	(-)	(-)	♰ death (respiratory failure)	persistence	N/A
28	Fontanella et al., 2020 [34]	67, M	PMBL	IV	almost at the carina	4	8 mos post-treatment	chemo (R-CHOP), RT	(-)	yes (esophagus)	(-)	(-)	CR	artificial closure	N/A
29	Wang et al., 2021 [35]	66, F	DLBCL	IV	trachea	35	20 days post-treatment	chemo (CVP)	(-)	(-)	(-)	(-)	good response → treatment discontinued per family's decision	persistence	N/A
30	Nwankwo et al., 2021 [36]	76, F	relapsed DLBCL	N/A	left bronchus	30	after discontinuation of chemo	chemo (tafasitamab-cxix/lenalidomide)	(-)	yes (esophagus)	(-)	(-)	respiratory failure, opted for palliative care	artificial closure	N/A
31	Hayashi et al., 2022 [37]	46, F	ALK-negative ALCL	IIE	trachea	N/A	after first CHOP cycle	chemo (CHOP, BV, GDP), ASCT	yes	(-)	(-)	yes	CR	artificial closure	2 yrs post-ASCT
32	Xuan et al., 2023 [38]	27, F	HL	III	trachea	20 x 12	after 2nd chemo cycle	chemo (ABVD)	(-)	yes (esophagus, trachea)	(-)	yes	remission	artificial closure	N/A
33	Ali et al., 2023 [39]	79, F	DLBCL	N/A	trachea	N/A	at diagnosis	none	(-)	(-)	yes	(-)	uncontrolled pneumonia and septic shock, opted for palliative care	artificial closure	N/A

In instances of lymphoma complicated by TEF/BEF, effective management of aspiration and infection is pivotal for improving outcomes. Among the documented cases, interventions targeted at fistula closure were administered in 22/33 cases: 8 underwent surgery, 9 received stent placement, 3 underwent a combination of stent and surgery, 1 received a combination of stent and OTSC (over-the-scope clip), and 1 was managed solely with OTSC. Notably, the prevalence of stent placements increased after 2010. Within these 33 cases, 6 were managed solely through enteral nutrition, out of which 4 achieved fistula closure through chemo/radiotherapy. While fistula management requires individualization, several reports recommend an initial conservative approach with enteral nutrition [9,10]. Furthermore, in cases where respiratory symptoms persist, less invasive interventions, such as removable stents, could be considered and escalated to surgical correction if warranted [11]. In our case, we opted for total parenteral nutrition and enteral nutrition without stent insertion because i) the esophageal stenosis was proximal and ii) inadequate stenosis within both the esophagus and trachea heightened the risk of stent migration. Given its potential to heal, adopting a conservative approach toward fistula management is advised. Moreover, as exemplified in our case, tailoring chemotherapy dosages to the patient's condition can ameliorate risks associated with bone marrow suppression and subsequent infections.

Two mechanisms have been proposed for the formation of lymphoma-related TEF/BEF [5]: the first attributes the fistula to primary lymphomatous involvement of the esophagus or tracheobronchial tree, whereas the second links it to the necrosis and breakdown of mediastinal lymph nodes between the esophagus and tracheobronchial tree. Specifically, the former can lead to fistula formation during the initial presentation, analogous to our case, whereas the latter, associated with lymph node necrosis, might result in fistula development during or after chemo/radiotherapy [12,13].

Our report categorizes cases into two types: those with a fistula present at the time of initial diagnosis or relapse, termed “pre-treatment fistula,” and those that emerge during or after chemo/radiotherapy, referred to as “post-treatment fistula.” Within the literature (Table 1), “pre-treatment fistula” was observed in 15/33 cases (45%), “post-treatment fistula” was noted in 17 cases (52%), and in one case, the fistula was identified posthumously. Additionally, “pre-treatment fistula” was more prevalent within Hodgkin's lymphoma cases (10/15 cases). Out of the 33 cases, information concerning the status of fistula closure was available for 31 cases. Notably, seven of these exhibited “healing” of the fistula (two of which had undergone stent placement prior to initiating treatment). Subsequently, the fistula closed, leading to the removal of the stent) [7,9,11,12,14-16]. All seven of these cases manifested a “pre-treatment fistula” and showcased a favorable response to chemotherapy and/or radiotherapy. In contrast, spontaneous closure was not observed when fistulas developed during or after therapy (“post-treatment fistula”), despite the lymphoma having entered remission, with such patients requiring interventions for fistula repair. Notably, the precise mechanism underlying fistula healing remains elusive; however, Rigney et al. proposed that esophageal defects undergo epithelialization, suggesting that the fistula might heal upon achieving sepsis control, positive tumor response to treatment, and the establishment of effective enteral nutrition [11]. Two out of the 15 “pre-treatment fistula” cases could not undergo lymphoma treatment due to respiratory failure or sepsis, which may suggest that fistula closure occurred more frequently in patients with relatively stable conditions.

In anatomical analyses, regarding fistula location, the majority were situated in the trachea, accounting for 64% (21/33) of the cases, followed by the left main bronchus (4 cases), and the right main bronchus (3 cases). One case involved both the trachea and left main bronchus, another occurred at the carina, and three were located distal to the right main bronchus. No significant differences were observed in fistula location between cases with healed versus persistent fistulas. Data on fistula size were available for 19 of the 33 cases, with a median diameter of 15 mm (range: 4-60 mm). Among these, two cases with sizes of 6 mm and 30 mm healed following chemotherapy. In our case, the fistula measured 5 × 10 mm, and no definitive correlation was observed between fistula size and closure.

Outcome data were available for 32 out of 33 cases. Of these, 20 cases survived without progression of the lymphoma, while 3 cases experienced progression or recurrence. In 4 cases, palliative care or treatment withdrawal was chosen, and 5 cases resulted in death where two due to respiratory failure, 2 from hemorrhage, and 1 from lymphoma progression. Among the seven cases where fistula healing was achieved, six had a median follow-up period of 12 months (range: 3-72 months), with no recurrence of either the lymphoma or the fistula (one case lacked a documented follow-up duration). Additionally, in our case, the patient remained progression-free for 46 months post-treatment, suggesting that long-term survival may be achievable when fistula healing is attained. In most instances, determining survival outcomes was challenging owing to the limited follow-up duration.

## Conclusions

TEF represents a rare complication within malignant lymphomas. The prognosis of TEF/BEF within lymphomas is more promising than that of solid tumors. Cases featuring initial fistula presentations have the potential for fistula resolution using chemotherapy and/or radiotherapy in tandem with effective management of aspiration and lymphoma. To investigate the mechanisms of fistula formation, healing, and long-term prognosis, it is necessary to accumulate additional cases and examine the characteristics of those with fistula complications.
